# Atlanta Academy of Medicine

**Published:** 1871-12

**Authors:** J. T. Johnson

**Affiliations:** Reporter


### ATLANTA ACADEMY OF MEDICINE.

J. T. JOHNSON, M.D., REPORTER.

OCTOBER 13th, 1871.

The President, Dr. J. P. Logan, in the chair.

Dr. W. M. Wells stated that he had recently witnessed the introduction of the probe into the cavity of the uterus by a Doctor who, as is known to many of the members, claims that the womb in the living body is seven or eight inches in its cavity, instead of two and a half or three, as taught by anatomists. The probe he uses is straight, and of the size of a crow-quill. He observed him carefully, and there is no doubt whatever as to its introduction to the depth of seven inches from the os tincæ, as he afterward measured the probe himself. Is sure that it was impossible for him to have perforated the fundus with the force used, the extremity of the probe being so blunt. Is not prepared to offer any explanation at this time. Intends to see other cases with him, and then to investigate the matter by attempting it himself when opportunity shall offer. The operator explains it by claiming that the womb is an erectile tissue, differing in the living woman from that found in the dissecting room. He holds that the probe, when introduced only three inches, or thereabouts, as we are in the habit of doing, reaches really only to the internal os—that is, passes only through the neck, not entering the cavity of the body at all. There is one thing that he admired—the instrument for applying caustic to the interior of the womb. Some 8 or 10 grs., he supposes, of the silver were powdered, and placed in a groove of a probe-shaped instrument. By turning this in the canula, he could bring the remedy in contact with every part of the cavity—an improvement, he must regard it, over the ordinary plan of the sponge or cotton, by which the most of the fluid is pressed out as it passes through the os.

Dr. W. F. Westmoreland has seen this gentleman introduce the probe in several instances; in one, as far as seven inches from the os tincæ. It was evident to him that he passed it through the Fallopian tube. The probe traverses the three inches through to the fundus without difficulty; then, by manipulation and pressing it to the side, he makes it pass two or three inches further, but with some difficulty, as though it were passing through some constriction. The patient experiences pain on the side toward which the instrument is turned. He has some difficulty in finding the orifice of the tube; has seen him make two or three failures in the attempt. He himself furnished a case: When the probe had passed one inch or more into the narrow or constricted part, the patient refused, from the excessive pain, to allow further manipulation; and himself interposed, fearing the consequences that might ensue. He has always witnessed a considerable shock during or following the operation—such as pallor, pain, sickness, and vomiting in one case.

While in New York, recently, he mentioned this practice to some of the prominent men, as Sims, Thomas, Nott. They agreed with him as to its passage through the tube. They know that it is impossible for the womb to be the length claimed, so much in excess of that found in the cadaver, as they have frequently observed it in the living body during operations, as ovariotomy, etc.

Agrees with Dr. Wells that his plan for introducing the caustic is a good one. But since the nitrate of silver is not always indicated, the using it in all cases would, of itself, mark his practice as empirical. He introduces the remedy every eight or ninth day. Thinks it is possible that some good may grow out of this, not as proving what the operator claims for the length of the uterus, but as showing the possibility of introducing an instrument through the Fallopian tube. Sims and others have accidentally accomplished this in exploring the fundus of the organ. This, in a few words, embraces his ideas on this question. As is known to most of the members, this Doctor is an irregular practitioner in this city.

Dr. W. H. Cumming.—Does he deny that he introduces it through the tube? I suppose he knows of the existence of the tubes?

Dr. Westmoreland.—He does deny it. He claims that the womb is an erectile tissue. This was an old idea of Rouget; it was exploded by ovariotomy. He contends that the neck of the womb is three inches and the body five, and that we make our applications, ordinarily, only to the neck.

Dr. Wells.—Recollecting the small diameter of the Fallopian tube, and its opening into the womb at right angles, it must seem impossible that a straight and stiff probe could be made to enter it; could not be done one time in ten, even if the womb were before you on the dissecting table. Would sooner think that he perforates the womb.

Dr. John Stainback Wilson.—Is the probe sharp-pointed?

Dr. Wells.—It is blunt, but rounded.

Dr. Wilson.—Must think, with Dr. Wells, that it would be difficult to pass it through the tube. May not the difficulty be thus explained? The vagina and womb are not on a straight line, but form an angle. The pushing upward of the womb would straighten this angle. Cannot this straightening, with some little stretching of the tissue of the womb itself, account for the unusual depth to which the probe is carried?

Dr. Wells.—The measurement was made from the os tincæ.

Dr. Wilson.—It seems that this explanation is more satisfactory than that of Dr. Westmoreland.

Dr. Wells.—Must think that Dr. Westmoreland's explanation is an impossible one.

Dr. W. F. Westmoreland.—It would be more easily accomplished with a straight and stiff probe than with one curved or flexible.

Dr. W. H. Cumming.—Would like to have the question referred to the Section on Anatomy, or to a special committee, for investigation as to the possibility of entering the Fallopian tube.

Dr. Harden is convinced that the probe passes into the Fallopian tube. Is satisfied, from examination of a recent work, that the tube passes from the womb at an obtuse angle, instead of at right angles, as he had heretofore supposed. The author states that a probe can be introduced without difficulty. We have been heretofore taught by anatomists that it is difficult to introduce even a bristle.

Dr. Bozeman.—Would ask Dr. Westmoreland if he could feel the point of the probe from the outside of the abdomen? and if it was in the medium line? Could not be exactly so, if through the tube. Has examined many wombs with many sounds, but never yet entered the tube—though he never tried, and never expects to, as he sees no good in so doing. Has seen a knife thrust into a man's body, but sees no good in so doing. Thinks that a probe, straight or slightly curved, could be so passed. As to the size of it, cannot see why one a fourth of an inch in diameter could not be so introduced. This seems large, but the ovum passes down through it. Remembers a case of tubal pregnancy—the only one he ever saw—which he will relate.

A negro woman was suddenly seized with excruciating pain in the abdomen, and other symptoms, that led at first to a diagnosis of colic. Enlargement of the abdomen and a sinking pulse indicated other trouble; decided that it was a case of tubal pregnancy. Death occurred the next morning, and the autopsy vindicated the correctness of the latter diagnosis. The abdomen was filled with blood; the Fallopian tube looked as if a dozen grains of powder had been exploded in it. By it was the ovum, one and a fourth inches in length, showing the amount of distension of which the tube is capable. Thinks that this operator introduces his probe through this tube.

Dr. Westmoreland.—Would reply to Dr. Bozeman, that he was not permitted to examine in the case in which the probe was carried to the depth of eight inches. But to a certain distance the probe is carried straight up, and then turned slightly aside. Must confirm Dr. Harden's idea of the anatomy of the womb and tube. Thinks, also, that it is easier to introduce the probe in the living than in the dead body.

Dr. Cumming's motion was carried, and a committee appointed, consisting of Dr. W. H. Cumming, W. F. Westmoreland, and W. M. Wells.

A motion to adjourn was defeated.

The regular subject for discussion, as selected at the last meeting, was "Blood-Letting."

Dr. Bozeman.—Will open the discussion himself, since Dr. Harden, the proposer of the question, will not. We well know that it is now regarded as improper to bleed under almost any circumstances, whereas many of us can remember when it was thought applicable to almost all inflammatory diseases. 'Tis not the object at present to discuss its applicability to special diseases, but merely the cause of the difference in the treatment: whether the type of disease itself has undergone a change, or science has improved so as to show that Rush and his disciples were wrong.

Dr. Harden.—There is a condition of apparent debility, accompanied by a depressed pulse, when the pulse will rise under the use of the lancet. Remembers the case of a patient that was once presented before the class in a feeble condition; he was bled again and again for some weeks, and recovered rapidly. A hasty view of the case would suggest his reconstruction by tonics, etc. He would not consider the congestions that the bleeding would relieve. Sydenham recommended bleeding in all congestions, even in the plague of London.

Dr. Wilson.—Was brought up to bleeding, but thinks the ultra bleeders went too far. He often used small bleedings in those cases described by Dr. Harden. On the other hand, for ten or fifteen years, has adopted the opposite plan of treatment, and has been just as successful. He now has no lancet. But there is one disease, acute pleurisy, in which he still adheres to the old plan; and if he should now treat a case of it, would hunt up his old lancet, else buy a new one.

Dr. Wells.—You can't buy a new one; at least, some time ago, one could not be found in the city.

Dr. Wilson.—Has been long willing to give it up, except in apoplexy and pleurisy.

Dr. Wells.—In speaking of congestion, if you alluded to that of pernicious fever, would ask what was the condition twenty-four hours after the bleeding, as compared to the moderate quinine treatment?

Dr. Wilson.—Just as good.

Dr. Bozeman.—Would not enter on the interminable field of special diseases, and the applicability to them of blood-letting, but whether diseases, as a class, are more asthenic now than forty years ago? Has no doubt himself but that they are. At the same time, a much nicer system of treatment prevails, and we have valuable remedies that were not then in existence. Take for example, bilious fever: We do not now think of bleeding, unless there is some complication; but years ago, when quinine and its value were unknown, venesection was the general practice. The difference in the treatment of disease generally, then and now, is accounted for both by this change of the type of disease and the advance of knowledge. The character of our country and the people, as the settlement has grown older, has also changed.

Dr. Cumming.—Would begin by criticising Dr. Bozeman's idea as to a change in the civilization of the country accounting for the change of disease and its treatment. This might hold good had not the same change worked in the old countries of Europe—as witness London, Paris, Berlin, etc. The opinion prevails there, also, that an asthenic type has come over disease, dating it from the advent of the cholera, in 1831. It is a mistake. It is not the diseases, but the physicians, that have changed. They know more now than then. If they of to-day could be transferred back thirty years, they would not bleed as then. Agrees with Dr. Harden, that we should oftener use the lancet than we now do. Remembers his own personal experience, having himself been bled three or four times, when it seemed that his body really must have cracked but for the venesection. It is important that this change-of-type theory be expunged from the mind of the profession. Veratrum in this country, and tartar emetic in Europe, can account to some extent for the change in the practice of bleeding.

Dr. Wells.—Agrees with Dr. Cumming as to the incorrectness of the change-of-type theory. The old practitioners had some excuse for resorting to blood-letting as they did, for they were not possessed of some of the fine sedatives of our day. Has never seen any cases calling decidedly for the lancet; apoplexy probably does. Has not bled more than a half dozen times in his practice.

Dr. W. F. Westmoreland.—One who boasts of having thrown away his lancet goes as far wrong as Cullen, who rode his hobby and caused the death of thousands. The middle ground is the right one. Cullen bled with a false idea of pathology. We are now likely to act on the other extreme. Would account for the change of treatment, first, by our improved knowledge of diseases and their pathology; again, we now have remedies to supply the place, to a degree, of blood-letting; again, must differ with Dr. Cumming, for he thinks that diseases *have* changed their character; and they change in the same locality, from year to year. There was a great change in Georgia during the years 1848 to 1853. When he commenced practice, he bled in pneumonia, but those years changed the treatment. Many of us know the character of that epidemic; everything was typhoid.

How common it was, fifteen or twenty years ago, to see reported in a journal forty or fifty cases of pneumonia treated by one physician, in which all recovered. In the practice of another physician, many died. They were probably equally intelligent. Then, how shall we explain it? One was in a locality where the sthenic type prevailed, as among the mountains; the other, perhaps, on the rice plantations of Georgia, or the prairies of Alabama, where the asthenic type prevailed. Fifty years ago, it was the practice to bleed at all times and stages of a disease. But it should be done in time to arrest the inflammation. It is of much service, used in proper time. But now we need not always resort to this remedy in cases that then would have demanded it, for we have a remedy in veratrum by which we can control the heart, and say how fast and how strong it shall beat.

Dr. Bozeman.—Would ask Dr. Cumming if it is more unreasonable that disease should change its character from the sthenic to the asthenic form, than that different epidemics of the same disease should present a different type? Has observed epidemics of scarlet fever when the lancet was necessary; in other epidemics—perhaps in two, three, four, or five years of the preceding one—the debility called for support of the system.

Dr. Cumming.—The fact of the change of treatment is not confined to one epidemic, or yet to Georgia or to South Carolina. The belief in the same change prevails in all the countries of Europe. They refer it to the advent of the cholera.

Dr. J. S. Wilson.—Agrees with Dr. Westmoreland and Dr. Cumming both. Thinks, with Dr. Cumming, that the theory of a general change from the sthenic to the asthenic, is a special plea set up to cover the bad treatment of former years, or to reconcile our apparent inconsistency. Thinks, with Dr. Westmoreland, that localities and different epidemic influences do exercise a modifying influence over disease.

Dr. Harden.—All seem to agree, at any rate, that we are now at the extreme opposite that of a few years ago.

